# Clinical features and high-risk indicators of central nervous system involvement in primary Sjögren’s syndrome

**DOI:** 10.1007/s10067-022-06448-w

**Published:** 2022-11-19

**Authors:** Wei Fan, Jennefer Par-Young, Kaiyan Li, Yi Zhang, Pingping Xiao, Li Hua, Lin Leng, Xuyan Chen, Richard Bucala

**Affiliations:** 1 Department of Rheumatology and Immunology, The Second Affiliated Hospital of Xiamen Medical College, Xiamen Medical College, Xiamen, 361021 China; 2 Department of Internal Medicine, Section of Rheumatology, Allergy & Immunology, Yale University School of Medicine, New Haven, CT 06520 USA

**Keywords:** Anti-SSA, Central nervous system, Complement C3, Glucocorticoids, Sjögren’s syndrome

## Abstract

**Background:**

Evidence for central nervous system involvement in primary Sjögren’s syndrome (pSS) patients is controversial and extremely limited. We aimed to describe the clinical profiles and high-risk indicators of primary Sjögren’s syndrome (pSS) patients with central nervous system (CNS) involvement (pSS-CNS).

**Methods:**

A total of 412 participants with pSS from a hospital in China from January 2012 to December 2019 were enrolled in the retrospective study. 42 pSS-CNS patients were compared with 370 pSS patients without CNS involvement. The clinical features, laboratory examinations, imaging characteristics, and treatment of the pSS-CNS cases were systematically analyzed. Potential risk factors related to pSS-CNS patients were identified by multivariate logistic regression analysis.

**Results:**

The prevalence of central nervous system involvement in the studied pSS patients was 10.2% (42/412), with 31.3% (14/42) of pSS patients having neurological manifestations as the initial symptom. The manifestations of hemiparesis (35.7%, 15/42), paraparesis (28.6%, 12/42), dysphonia (31.0%, 13/42), blurred vision (21.4%, 9/42), and dysfunctional proprioception (23.8%, 10/42) were more common in the pSS-CNS patients. Cerebral infarction (57.1%, 24/42), demyelination (31.0%, 13/42), myelitis (23.8%, 11/42), and angiostenosis (21.4%, 9/42) were most often found on MRI or CT scan imaging in the pSS-CNS patients. Intrathecal IgG level and total protein of cerebrospinal fluid were increased in 50% (8/16) of the pSS-CNS group. In comparison with patients without CNS involvement, the pSS-CNS patients were found to also have kidney and lung involvement, hematologic abnormalities, positive ANA and anti-SSA antibody tests, and reduced complement 3 (C3) and complement 4 (C4) levels (all *p* < 0.05). The prevalence of lung involvement, immune thrombocytopenia, and high-titer ANA (1:1000) were significantly higher in pSS-CNS disease activity compared to those in the moderately active group. Multivariate analysis identified lung involvement, anti-SSA positivity, and low C3 levels as prognostic factors for pSS-CNS. After high-dose glucocorticoids and immunosuppressive therapy, 60.5% (26/38) of pSS-CNS patients improved, 36.8% (14/38) were unresponsive to treatment, and 2.6% (1/38) died.

**Conclusion:**

Clinical features are diverse in pSS-CNS patients, and the morbidity rate is low. CNS involvement was the initial presentation in state percentage here pSS patients. Pulmonary involvement, a positive anti-SSA antibody test, and reduced C3 levels are potential risk factors for CNS involvement in pSS. Treatment with high-dose glucocorticoids and immunosuppressive therapy appeared effective in 60% of pSS-CNS patients.

**Table:** 

**Key Points** *• The CNS manifestations of pSS are diverse, and CNS imaging and CSF analysis are important for the diagnosis.* *• Pulmonary involvement, positive anti-SSA, and reduced C3 levels are potential risk factors of pSS-CNS.* *• About 60% of pSS-CNS patients were responsive to high-dose glucocorticoid administration and immunosuppressive therapy.*

## Introduction

Sjögren’s syndrome (SS) is a chronic autoimmune inflammatory disease characterized by the presence of lymphocytic infiltrates in the exocrine glands. It commonly causes dry mouth (xerostomia) and dry eyes (xerophthalmia), but multiple organs can be affected by the extraglandular manifestations of this disease within the musculoskeletal, renal, nervous, pulmonary, and hematological systems [1–3]. In the absence of an underlying associated rheumatic disease, the condition is called primary Sjögren’s syndrome (pSS) [4]. Neurological complications of Sjögren’s syndrome may involve both the central (CNS) and peripheral nervous systems (PNS) [5, 6]. The clinical features of neurologic pathology of Sjögren’s syndrome are diverse and can precede the typical glandular manifestations, leading to features that may obscure early diagnosis. Indeed, some pSS patients can experience severe central neuropathy as the initial symptom [7, 8]. If diagnosis and treatment are not timely, neurologic disease may lead to residual dysfunction and permanent disability, leading to continuing challenges in the management of CNS involvement in pSS patients [9, 10].

In the present report, we describe the clinical presentation and laboratory findings including cerebrospinal fluid (CSF) analysis, imaging characteristics, treatments, and outcomes in 42 pSS-CNS patients compared with 370 pSS patients without CNS involvement. We also analyzed potential risk factors that may be useful to identify CNS involvement in pSS patients and to guide further clinical studies.

## Materials and methods

### Study population and clinical data

A total of 412 patients admitted to the Second Affiliated Hospital of Xiamen Medical College from January 2012 to December 2019 who met the 2002 international classification criteria for Sjögren’s syndrome as proposed by the American-European consensus group were investigated [11]. Within the study cohort, 42 pSS patients had CNS involvement based on assessment by a rheumatologist and a neurologist; another 370 pSS were without CNS involvement. Patients with additional connective tissue diseases (secondary SS) were excluded from study. The study was performed according to the current National Health and Family Planning Commission of China ethical standards and was reviewed and approved by the hospital ethics committee.

For the 412 pSS patients, demographics (age and gender) as well as clinical data (disease duration, oral and ocular dryness, fever, and symptoms involving the joints, skin, pulmonary, circulatory, and renal systems) were collected. In addition, pSS-CNS clinical records included predisposing factors, and imaging characteristics, CSF examination, treatment, and outcome were analyzed.

### Assessment of central nervous system involvement

For the CNS domain of the EULAR Sjögren’s Syndrome Disease Activity Index (ESSDAI), the evaluation of neurological disease activity is divided into three levels and scored as 0 (no activity), 10 (moderate activity), or 15 (high activity). The three levels are, respectively, absence of currently active CNS involvement, moderately active CNS features (e.g., cranial nerve involvement of central origin, optic neuritis, or multiple sclerosis-like syndrome with symptoms restricted to pure sensory impairment or confirmed cognitive impairment), and highly active CNS features ( e.g., cerebral vasculitis with cerebrovascular accident or transient ischemic attack, seizures, transverse myelitis, lymphocytic meningitis, and multiple sclerosis-like syndrome with motor deficit) [12]. We reviewed the medical records of patients with any of these CNS features, laboratory and cerebrospinal fluid analyses, and imaging studies (CT scan, MRI).

### Laboratory data

Laboratory data were recorded in all patients. Blood counts, biochemical tests, erythrocyte sedimentation rate (ESR), and C-reactive protein (CRP) were measured by automated instrumentation. The immunological tests including anti-SSA, anti-SSB, and anti-AQP4 antibody levels were performed using an immunoblotting method; antinuclear antibodies (ANA) were tested by IIF (indirect immunofluorescence) using HEp-2 cell substrate, immunoglobulin (Ig) A, IgG, IgM, complement 3 (C3), and complement 4 (C4); rheumatoid factor (RF) and CSF examination were measured with a chemical luminescence immunity analyzer.

### Statistical methods

The data were analyzed using SPSS Version 22.0 (SPSS Inc., Chicago, IL, USA) and R version 3.6.3. The Kolmogorov–Smirnov test was performed to determine if the data were normally distributed. Continuous data are presented as mean ± standard deviation (SD), continuous data of non-normal distribution are presented as median with 95% CI, and numbers and percentages (%) are indicated for categorical variables. The continuous variables were analyzed using Student’s t-test and/or Mann–Whitney U-test depending on the normality of the distribution. The comparisons of the categorical variables were performed by Fisher’s exact tests. Univariate and multivariate analyses were used to examine correlations between risk factors and central neuropathy. The receiver operating characteristic (ROC) and calibration curves were plotted using the “pROC” (v1.16.1) and “rms” (v5.1–4) packages, respectively. The calibration curve was plotted with 1,000 times bootstrap resampling using “calibrate” function in the R package “rms.” The calibration performance of the model was tested by the Hosmer–Lemeshow goodness-of-fit test using R package “Resource Selection” (v0.3–5) [13]. For each comparison, a *p* value < 0.05 was considered statistically significant.

## Results

### The characteristics of pSS patients with or without CNS involvement

The demographic data, clinical features, and laboratory tests collected from 42 pSS-CNS patients with and 370 without CNS involvement are presented in Table 1. The female to male ratio was 7.2:1, the mean age at was 48.48 ± 16.18, and the mean disease duration was 36.85 (range: 31.0—48.7) months in 412 pSS patients. Compared with pSS patients without CNS involvement, those with CNS involvement showed higher prevalence of lung involvement (28.6% vs. 15.4%, *p* < 0.05), renal involvement (9.52% vs 0.2.4%, *p* < 0.05), ANA (95.2% vs. 71.1%, *p* < 0.05), anti-SSA positive (88.1% vs. 58.9%, *p* < 0.05), and lower levels of C3 (0.88 ± 0.30 vs. 1.05 ± 0.32, *p* < 0.05) and C4 (0.18 ± 0.06 vs. 0.23 ± 0.09, *p* < 0.05). Hematological abnormalities also were significantly more frequent in those with CNS involvement (73.8 vs. 56.8, *p* < 0.05), although comparison of individual components of the hemogram, i.e., the levels of hemoglobin, WBCs, and platelet counts did not show significant differences between the two groups. With respect to comparisons between high CNS disease activity group and the moderate activity group, there were increased frequencies of lung involvement (0% vs. 12%, *p* < 0.05), high-titer (1:1000) ANA (11.1% vs. 36.4%, *p* < 0.05), and immune thrombocytopenia (218.11 ± 68.47 versus 137.94 ± 84.67, *p* < 0.05). This comparison is presented in Table 2. Additionally, five out of pSS-CNS patients were tested for NMO-IgG (anti-AQP4 antibody), and two were positive.

**Table 1:** Demographic, clinical features, and laboratory measures of primary Sjögren’s syndrome patients with or without central nervous system involvement

Characteristic	With CNS involvement (*N* = 42)	Without CNS involvement (*N* = 370)	*p* value
Sex, female *n* (%)	38 (90.5)	324 (87.6)	0.803
Age (mean ± SD), years	49.50 ± 16.67	48.36 ± 16.14	0.667
Disease duration, months	40.6 (26.6, 57.0)	36.4 (30.3, 46.4)	0.668
Xerostomia, *n* (%)	36 (85.7)	284 (76.8)	0.241
Xerophthalmia, *n* (%)	20 (47.6)	153 (41.4)	0.510
Arthralgia, *n* (%)	19 (45.2)	160 (43.2)	0.870
Skin rash, *n* (%)	11 (26.2)	96 (25.9)	1.000
Fever, *n* (%)	5 (11.9)	89 (24.1)	0.083
Hematologic changes, *n* (%)	31 (73.8)	210 (56.8)	0.046
Lung involvement, *n* (%)	12 (28.6)	57 (15.4)	0.047
Kidney involvement, *n* (%)	4 (9.52)	9 (2.4)	0.034
ANA positive, *n* (%)	40 (95.2)	263 (71.1)	0.000
Anti‐SSA positive, *n* (%)	37 (88.1)	218 (58.9)	0.000
Anti‐SSB positive, *n* (%)	13 (31.0)	109 (29.5)	0.859
RF > 20 IU/ml, *n* (%)	19 (45.2)	132 (35.7)	0.239
ESR > 20 mm/h, *n* (%)	23 (54.8)	243 (65.7)	0.175
CRP > 8 mg/L, *n* (%)	7 (16.7)	83 (22.4)	0.554
C3 (mean ± SD), g/L	0.88 ± 0.30	1.05 ± 0.32	0.002
C4 (mean ± SD), g/L	0.18 ± 0.06	0.23 ± 0.09	0.000
IgG (mean ± SD), mg/mL	15.78 ± 4.46	17.46 ± 7.41	0.135
IgA (mean ± SD), mg/mL	2.93 ± 1.80	3.00 ± 2.32	0.684
IgM (mean ± SD), mg/mL	1.37 ± 0.95	1.46 ± 1.14	0.637
WBC (mean ± SD), × 10^9^/L	6.27 ± 4.72	5.80 ± 3.46	0.423
Hb (mean ± SD), g/L	113.64 ± 17.76	112.20 ± 22.17	0.628
PLT < 100 × 10^9^/L, *n* (%)	12 (28.6)	201 (27.6)	0.875
PLT < 30 × 10^9^/L, *n* (%)	30 (8.1)	5 (11.9)	0.383
ALT (mean ± SD), u/L	40.56 (33.85, 47.82)	36.08 (27.72, 45.21)	0.678
AST (mean ± SD), u/L	40.91 (35.85, 46.47)	36.02 (24.60, 50.06)	0.559

ANA, antinuclear antibodies; C3, complement component 3; C4,complement component 4; CRP, C-reactive protein; ESR, erythrocyte sedimentation rate; RF, rheumatoid factor; IgA, immunoglobulin A; IgG, immunoglobulin G; IgM, immunoglobulin M; WBC, white blood cell; Hb, hemoglobin; PLT platelet; ALT, alanine transaminase; AST, aspartate aminotransferase

**Table 2:** Demographic, clinical features, and laboratory data comparison of the moderately active and highly active patient groups with CNS-pSS

Characteristic	Moderately active group (*N* = 9)	Highly active group (*N* = 33)	*p* value
Sex, female, *n* (%)	9 (100)	28 (84.8)	1.000
age (mean ± SD), years	48.78 ± 16.52	49.70 ± 16.97	0.886
Disease duration, months	63.11 (18.4, 121.2)	34.48 (21.8, 47.6)	0.307
Xerostomia, *n* (%)	8 (88.9)	28 (84.8)	1.000
Xerophthalmia, n (%)	5 (55.6)	15 (54.5)	0.714
Arthralgia, n (%)	2 (22.2)	17 (51.5)	0.149
Skin rash, *n* (%)	0 (0)	11 (33.3)	0.083
Fever, *n* (%)	2 (22.2)	3 (9.1)	0.288
Lung involvement, *n* (%)	0 (0.0)	12 (36.4)	0.041
Kidney involvement, *n* (%)	0 (0.0)	4 (12.1)	1.000
ANA (1:1000) positive, *n* (%)	1 (11.1)	18 (54.5)	0.027
Anti‐SSA positive, *n* (%)	8 (88.9)	29 (87.9)	1.000
Anti‐SSB positive, *n* (%)	1 (11.1)	12 (36.4)	0.232
RF positive, *n* (%)	3 (33.3)	16 (48.5)	0.477
ESR > 20 mm/h, *n* (%)	5 (55.6)	18 (54.5)	1.000
CRP > 8 mg/L, *n* (%)	2 (22.2)	5 (15.2)	0.631
C3 (mean ± SD), g/L	0.81 ± 0.19	0.91 ± 0.32	0.387
C4 (mean ± SD), g/L	0.17 ± 0.06	0.18 ± 0.07	0.575
IgG (mean ± SD), mg/mL	14.5 ± 5.23	16.1 ± 4.26	0.135
IgA (mean ± SD) mg/mL	1.98 ± 1.78	3.19 ± 1.75	0.684
IgM (mean ± SD), mg/mL	0.91 ± 0.53	1.50 ± 1.01	0.460
WBC (mean ± SD), × 10^9^/L	6.41 ± 2.55	6.23 ± 5.19	0.385
Hb (mean ± SD), g/L	115.33 ± 9.89	112.58 ± 19.49	0.975
PLT < 100 × 10^9^/L, *n* (%)	0 (0.0)	12 (36.4)	0.041
ALT (mean ± SD), u/L	23.47 ± 15.34	39.52 ± 33.28	0.668
AST (mean ± SD), u/L	18.11 ± 8.65	40.90 ± 40.76	0.624
ALB albumin	35.41 ± 3.75	36.76 ± 4.18	0.385

ANA, antinuclear antibodies; C3, complement component 3; C4, complement component 4; CRP, C-reactive protein; ESR, erythrocyte sedimentation rate; RF, rheumatoid factor; IgA, immunoglobulin A; IgG, immunoglobulin G; IgM, immunoglobulin M; WBC, white blood cell; Hb, hemoglobin; PLT, platelet; ALT, alanine transaminase; AST, aspartate aminotransferase

### The neurological manifestations of CNS involvement in primary Sjögren’s patients

The prevalence of CNS involvement in all subjects was 10.2% (42/412), and 31.3% (14/42) cases of those with pSS-CNS showed neurological manifestations as the initial symptom. Predisposing factors were not clear in most patients, but 7.1% (3/42) of subjects had disease-related fatigue. The common neurological manifestations were hemiparesis (35.7%, 15/42), paraparesis (28.6%, 12/42), dysphonia (13/42, 31.0%), dysfunctional proprioception (10/42, 23.8%), blurred vision (21.4%, 9/42), headaches with imaging abnormalities (14.2%, 6/42), cognitive impairment (9.5%, 4/42), central facial paralysis (9.5%, 4/42), syncope (2.3%, 1/42), and urinary disorders (2.3%, 1/42). All the pSS-CNS patients underwent neurologic imaging examination, which showed multifocal lesions. Cerebral infarction (57.1%, 24/42), myelitis (57.1%, 11/42), demyelination (31.0%, 13/42), and angiostenosis (21.4%,9/42) were the most common MRI or CT scan findings in the in pSS-CNS patients. The lesions in 42 pSS-CNS patients were mainly localized in the lateral cerebral ventricles (31.0%, 13/42), frontoparietal lobes (28.5%, 12/42), basal ganglia (26.2%, 11/42), corona radiata (26.2%, 11/42), thoracic cord (19.0%, 8/42), and carotid, vertebral, and cerebral arteries (23.8%, 10/42). Cerebrospinal fluid examination was performed in 16 pSS-CNS patients, 1 case (6.3%) was normal, and the remaining 15 cases (93.7%) showed abnormal cerebrospinal fluid indicators, including pleocytosis in 6 cases (37.5%), elevated intrathecal antibody synthesis of IgG in 8 cases (50.0%), elevated CSF total protein in 8 cases (50%), increased percentage of lymphocyte in 7 cases (43.8%), and increased percentage of neutrophil 5 cases (31.3%). The CSF glucose and chloride values were within normal limits in sixteen patients (Tables 3, 4 and 5).

**Table 3:** Neurological manifestations of primary Sjögren’s syndrome patients

Neurological manifestations	Number	Percentage (%)
Hemiparesis	15	35.7
Paraparesis	12	28.6
Blurred vision	9	21.4
Dysphonia	13	31.0
Dysfunctional proprioception	10	23.8
Limb numbness	6	14.2
Headaches with imaging Abnormalities	6	14.2
Cognitive impairment	4	9.5
Aseptic meningitis	2	4.8
Central facial paralysis	4	9.5
Syncope	1	2.3
Urination disorders	1	2.3

**Table 4:** Imaging features of central nervous system in Sjögren’s syndrome patients

Imaging features	Number	Percentage (%)
**CNS radiological results**		
Cerebral infarction	24	57.1
Demyelination	13	31.0
Myelitis	11	26.2
Angiostenosis	9	21.4
Brain atrophy	8	19.0
Leukoaraiosis	4	9.5
Cerebral ischemia	4	9.5
Cerebral hemorrhage	2	4.7
**Location of lesion**		
Lateral cerebral ventricle	13	31.0
Frontoparietal	12	28.5
Basal ganglia	11	26.2
Corona radiata	11	26.2
Carotid, vertebral, and cerebral arteries	10	23.8
Brain atrophy	7	16.7
Brain cortex	4	9.5
Cervical cord	7	16.7
Thoracic cord	8	19.0
Lumbar cord	3	7.2
Cerebellum	4	9.5
Thalamus	4	9.5
Brain stem	1	2.3
Medulla	1	2.3

**Table 5:** Cerebrospinal fluid (CSF) features of primary Sjögren’s syndrome patients with central nervous system involvement

CSF features	Number (16)	Percentage (%)
Pleocytosis	6	37.5
Levels of neutrophils elevated	5	31.3
Levels of lymphocytes elevated	7	43.8
Intrathecal antibody synthesis of IgG	8	50.0
Total protein (mg/L)	8	50.0

CSF, cerebrospinal fluid, IgG, immunoglobulin G

### Specific factors associated with CNS involvement in Sjögren’s syndrome

To assess potential risk factors for CNS involvement in pSS, we examined a series of indicators commonly used in clinical practice first by univariate analysis and then multivariate logistic regression analysis. As is shown in Table 6, pulmonary and renal involvement, hematologic changes, ANA and anti-SSA autoantibodies, and C3 and C4 were found to be associated with CNS involvement, and these factors were included in the assessment. Lung involvement (OR = 2.50, 95% CI 1.11–5.59, *p* = 0.026), the presence of anti-SSA autoantibodies (OR = 3.63, 95% CI 1.12–11.8, *p* = 0.032), and low level of C3 (OR = 2.3, 95% CI 1.03–5.14, *p* = 0.043) were found to be significantly different between pSS patients with and without CNS involvement. Other factors were not observed to be significantly related to the development of CNS involvement in pSS patients.

**Table 6:** Univariate and stepwise multivariate analysis of risk factors for CNS involvement in primary Sjögren’s syndrome

Characteristics	Univariate analysis			Multivariate analysis		
	Odds ratios	95% CI	*p* value	Odds ratios	95% CI	*p* value
Hematologic changes, *n* (%)	2.15	1.05–4.40	0.037	-		
Lung involvement, *n* (%)	2.20	1.06–4.54	0.034	2.50	1.11–5.59	0.026
Kidney involvement, *n* (%)	4.22	1.24–14.4	0.021	-		
ANA positive, *n* (%)	8.14	1.93–34.3	0.004	-		
Anti‐SSA positive, *n* (%)	6.62	2.32–18.9	0.000	3.63	1.12–11.8	0.032
RF positive, *n* (%)	1.49	0.78–2.84	0.225	-		
C3 < 0.8 g/L	4.26	2.21–8.20	0.000	2.30	1.03–5.14	0.043
C4 < 0.2 g/L	3.52	1.79–6.92	0.000	-		
PLT < 30 × 10^9^/L, *n* (%)	1.53	0.56–4.19	0.406	-		

ANA, antinuclear antibodies; C3, complement component 3; C4, complement component 4; RF, rheumatoid factor; PLT, platelet

### Establishment and evaluation of risk prediction models for CNS involvement in Sjögren’s syndrome

**Fig. 1:**
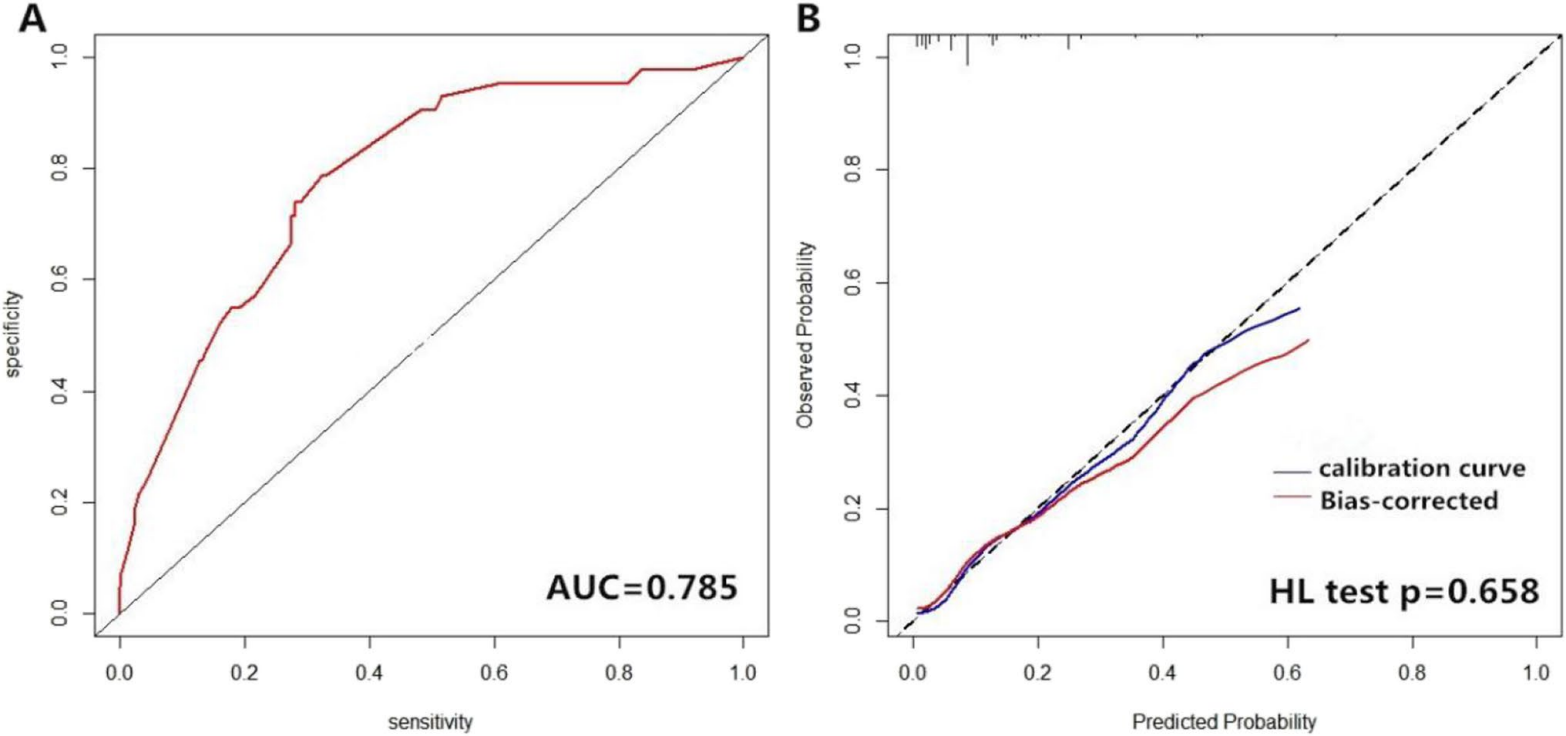
Evaluation of the performance of the risk prediction model. (A) Receiver operating characteristic (ROC) curve of the prediction model. The AUC was 0.785. (B) Calibration curve for the logistic regression model. The calibration curve was plotted with 1,000 times bootstrap resampling. The *p* value was calculated with Hosmer–Lemeshow (HL) goodness-of-fit test (*p* = 0.658). The solid blue line represents original calibration curve and the blue bands represent bias-corrected calibration curve

We carried out ROC and calibration curve analyses to evaluate the performance of the risk prediction model. The AUC was 0.785 (95% confidence interval, 0.716–0.854). The calibration plot showed relatively high agreement between the actual observation and the prediction by the model in terms of CNS involvement rate, and the Hosmer–Lemeshow goodness-of-fit test had a value of *p* = 0.658, suggesting an appreciable discrimination and calibration performance of the prediction model (Fig. 1).

### Treatment with CNS involvement in Sjögren’s syndrome

All patients were treated with glucocorticoids at different doses in accord with disease severity. Of the 42 patients, 30 (71.4%) were given immunomodulating and immunosuppressive drugs; 29 (69.0%) were given high-dose methylprednisolone (> 1 mg/kg), 12 of which were given in combination with intravenous immunoglobulin (IVIG); and 13 patients (31.0%) received oral prednisone (≤ 40 mg/d). Clinical benefit with documented improvement of motor and sensory function or stabilization of status upon evaluation by a physician was considered as favorable responses. In the 38 patients that underwent evaluation, 23 (60.5%) were found to have a favorable response after treatment, among which the recurrence rate was 39.5% (15/38). Fourteen patients (14/38, 36.8%) were unresponsive, and 1 patient (2.6%) died. Of three patients who failed to respond to combination treatment with glucocorticoids and immunosuppression, one patient improved after treatment with immunoadsorption.

## Discussion

Primary Sjögren’s syndrome is a chronic autoimmune inflammatory disease that can affect the exocrine glands and different organ systems, including the nervous system. The CNS manifestations of pSS are of unclear pathogenesis and the object of increasing attention. It has been reported that the incidence of central neurological symptoms of pSS ranges from 2.5% to 60%, with the widely reported range arising from a lack of unified diagnostic criteria, variations in the nature of the manifestations considered, and the patient recruitment modalities [14–18]. Guillermo et al. [5] concluded that the high prevalence of pSS-CNS involvement in their neuropsychiatry study is probably attributable to the broad definition of manifestations, such as fatigue and headache which are common in pSS. Fan et al. [14] and Flores-Chávez et al. [19] reported that the incidence of CNS involvement in pSS patients was 9.8% and 13%, respectively. In the present study, the prevalence of CNS involvement was identified at 10.1% of subjects, which was consistent with the aforementioned studies. The clinical phenotype of CNS complications in pSS was quite variable. The most common in our study was hemiparesis, paraparesis, dysphonia, dysfunctional proprioception, or blurred vision. Some patients presented with dizziness, headache, and cognitive impairments. pSS is more common in females, and the female to male ratio in pSS-CNS patients in this cohort was 7.4:1. Among this group, 31.3% (14/42) of the patients have CNS complications as initial symptoms, which may be ignored, leading to delayed diagnosis and treatment. In one study, neurological manifestations occurred in one-third of cases before the diagnosis of pSS, and two-thirds of cases were present at the time of diagnosis [18]. DeLaland et al. [20] proposed that women with unexplained CNS manifestations should be more closely evaluated for the possibility of Sjögren’s syndrome.

ESSDAI is a widely used assessment tool of disease activity and prognosis for pSS patients in clinical studies [19, 21–23]. CNS or PNS manifestations were suggested to be associated with greater pSS activity and other systemic manifestations of pSS according to ESSDAI values [5]. We confirmed that the group with CNS involvement versus non-CNS involvement had a significantly higher proportion of patients with hematologic changes and lung and kidney involvement. The frequencies of lung involvement and immune thrombocytopenia were significantly higher in the high disease activity group of pSS-CNS than the moderate group as assessed using CNS domain of ESSDAI. However, in the multiple logistic regression model, only lung involvement was retained as a significant and independent risk factor related to CNS complications (OR = 2.50, *p* = 0.026). Previous studies also suggested an association between neurological involvement and pulmonary involvement, although the pathogenic relationship is unclear [10]. The main pathogenic mechanism may be related to the disease-related inflammatory response, with interactions between the innate and adaptive immune system playing a cardinal role [24, 25].

High titer ANA positivity (1:1000) was not confirmed as a feature directly correlated with the CNS involvement activity of pSS patients in previous studies, while anti-SSA autoantibodies and low levels of C3/C4 were more frequently observed in patients with neurological involvement [3, 10, 18, 21, 26, 27]. In the present study, anti-SSA positivity and reduced C3 levels were confirmed to be potential risk factors for CNS involvement by multivariate analyses, and this was consistent with previous studies [26, 27]. Regarding pathogenic factors, some researchers have suggested that CNS injury is related to the presence of ANA and anti-SSA autoantibodies. Evidence of an immunologically-mediated mechanism further is supported by the demonstration of intrathecal activation of the terminal complement pathway [26, 28–30]. However, even if anti-SSA autoantibodies were more likely to be present in the pSS-CNS group, there were no differences between the high and moderate activity groups of pSS-CNS patients. In fact, a recent analysis showed that anti-Ro/SSA positive patients were characterized by a higher frequency of activity in many ESSDAI domains except for the PNS and CNS ones [31]. Baldin et al. showed that single laboratory abnormalities had limited or poor prognostic value in pSS associated with severe extraglandular features, but patients who had two or more adverse serological values were more likely to develop severe systemic complications with worse outcome and consequently required more aggressive treatment [3]. The presence of anti-aquaporin-4 autoantibodies (AQP4-IgG) may be directly pathogenic and related to CNS disease in pSS [9, 15, 32]. More than 80% of pSS patients with acute neurological events such as myelitis or optic neuritis are reported to be AQP4-IgG positive [33]. We measured NMO-IgG (anti-AQP4 autoantibody) in five patients suspected of neuromyelitis optica spectrum disorders and found two patients be positive for this autoantibody.

CNS imaging and CSF analysis are important for the diagnosis of autoimmune-mediated neurological diseases [28, 34, 35]. In the present study, all patients exhibited brain imaging abnormalities and variation with multiple focal lesions. Some patients also showed visible vascular stenosis, the underlying cause of which remains unclear. On CSF analysis of 16 patients in this study, there was pleocytosis in 6 patients (37.5%) and increased intrathecal IgG levels in 8 patients (50.0%). Pars et al. [34] showed that elevated intrathecal IgG and pleocytosis occurred in 25% of patients with CNS involvement. Elane et al. suggested that in SS patients with active CNS involvement, there was an increase in white blood cell counts in 50% of patients and increased intrathecal IgG concentrations in 43% [36]. Recently, Tjensvoll et al. [37] reported anti-NR2 antibodies in CSF as a new marker that correlates with CNS impairment and that may be a pathogenetic factor for neuronal dysfunction in pSS patients. This observation further supports the recommendation that examination of cerebrospinal fluid and CNS imaging is important for the diagnosis and evaluation of pSS-CNS disease activity.

At present, there are no consensus guidelines for the treatment of pSS-CNS patients. In our study, we found that in pSS-CNS patients with high disease activity, rapidly progressing lesions, and severe symptoms, glucocorticoids combined with immunosuppressive treatment give favorable results. Intravenous cyclophosphamide pulses also are frequently recommended [6]. There are also reports of methotrexate, cyclosporine, azathioprine, rituximab, and IVIG as effective adjunctive treatment in some patients. Plasmapheresis with immunosuppressive treatment also has been reported to have clinical benefit [8, 38–40]. These treatments nevertheless do not completely reverse CNS disease, but provide varying different degrees of improvement in symptoms. The present study included one example of immunosorbent therapy for a patient who failed to respond to the above treatments, which with some measure of efficacy. Further studies are clearly indicated to evaluate effective therapies.

There are some limitations in this study. First, the patients were not ethnically diverse. The limited pSS-CNS sample size of our cohort and the single-center study design reduced statistical power for more definitive conclusions. Second, the data were evaluated retrospectively and some examinations were not available for all patients, leaving the possibility of reporting bias. Additionally, there are no systematic scores applied for evaluating the efficacy of treatment. A larger prospective study is needed to both confirm and extend the present findings toward the ultimate goal of more effective evaluation and management of neurologic manifestations in pSS.
