# Correction to: Clinical features and high-risk indicators of central nervous system involvement in primary Sjögren’s syndrome

**DOI:** 10.1007/s10067-022-06488-2

**Published:** 2022-12-24

**Authors:** Wei Fan, Jennefer Par-Young, Kaiyan Li, Yi Zhang, Pingping Xiao, Li Hua, Lin Leng, Xuyan Chen, Richard Bucala

**Affiliations:** 1 Department of Rheumatology and Immunology, The Second Affiliated Hospital of Xiamen Medical College, Xiamen Medical College, Xiamen, 361021 China; 2 Department of Internal Medicine, Section of Rheumatology, Allergy & Immunology, Yale University School of Medicine, CT 06520 New Haven, USA

**Correction to: Clinical Rheumatology**

**https://doi.org/10.1007/s10067-022-06448-w**

In the original published version of the above article contained incomplete information. The funding section has been corrected as follows:

Funding This work was supported by the National Natural Science Foundation of China for youth project (Grant No. 81901669/National Natural Science Foundation of China for Youth Project), Xiamen Science and Technology (Medical and Health) Project (No. 3502Z20184061), Xiamen Medical and Health Guidance Project (Nos. 3502Z20179008 and 3502Z20199137), and Fujian Medical and Health Training Project for young and middle-aged backbone talents (No. 2020GGB068).

The original article has been corrected.

